# Fabrication of a form- and size-variable microcellular-polymer-stabilized metal nanocomposite using supercritical foaming and impregnation for catalytic hydrogenation

**DOI:** 10.1186/1556-276X-7-283

**Published:** 2012-05-31

**Authors:** Weisheng Liao, Ben-Zen Wu, Hungchi Nian, Hsiang-Yu Chen, Jya-Jyun Yu, KongHwa Chiu

**Affiliations:** 1 Department of Chemistry, National DongHwa University, Shoufeng, Hualien, 97401, Taiwan; 2 Department of Chemistry, Chung Yuan Christian University, Chung-Li, Tao-Yuan, 32023, Taiwan; 3 Department of Environmental Engineering and Science, Feng Chia University, Taichung, 40724, Taiwan

**Keywords:** Nanoparticle, Heterogeneous catalysis, Supercritical fluids, Foaming, Impregnation

## Abstract

This article presents the fabrication of size-controllable and shape-flexible microcellular high-density polyethylene-stabilized palladium nanoparticles (Pd/m-HDPE) using supercritical foaming, followed by supercritical impregnation. These nanomaterials are investigated for use as heterogeneous hydrogenation catalysts of biphenyls in supercritical carbon dioxide with no significant surface and inner mass transfer resistance. The morphology of the Pd/m-HDPE is examined using scanning electron microscopy images of the pores inside Pd/m-HDPE catalysts and transmission electron microscopy images of the Pd particles confined in an HDPE structure. This nanocomposite simplifies industrial design and operation. These Pd/m-HDPE catalysts can be recycled easily and reused without complex recovery and cleaning procedures.

## Background

Nanotechnology has resulted in research that examines a scale of matter typically between 1 and 100 nm wherein properties are dependent on the size and form. The greater surface area per mass compared with larger particles with identical chemical composition makes nanomaterials an excellent catalyst and also makes them more biologically active. When examining possible exposure routes for manufactured nanoparticles or during their production and inhalation, oral and dermal exposure are the most obvious, depending on the type of product in which nanoparticles are used. Hence, the development of green methods for the fabrication of nanomaterials has become increasingly relevant as chemists look to shape a more sustainable future.

Heterogeneous catalytic hydrogenation in supercritical fluids, especially in supercritical carbon dioxide (sc-CO_2_), has recently become an active research area because of its advantages over conventional organic-solvent-based systems. Sc-CO_2_ has proven to be an effective solvent for a wide range of homogeneous transition-metal-catalyzed reactions. The readily achievable critical point of sc-CO_2_ (*T*c = 31.1 ***°***C, *P*c = 73.7 bar) is one of its many advantages, which also include its low cost, low toxicity, no flammability, and the ability to tune the reactive properties by small variations, i.e., variation in temperature or pressure in the sc-CO_2_ density [1]. Their advantages also include enhanced mass and heat transfer, adjustable salvation ability, total H_2_ miscibility, elimination of the gas/liquid interface, extended catalyst lifetime, ease of separation from products, and minimal organic solvent waste [2-4]. Transition metals (e.g., Pd, Rh, Ru, and Pt) supported on various matrices (e.g., activated carbon, Al_2_O_3_, SiO_2_, SBA-15, and MCM-48) have been prepared and applied for catalytic hydrogenation of various reactants in supercritical fluids.

Previous research showed that nanosized palladium particles (diameter = 2 to 10 nm) could be deposited uniformly in the interior of high-density polyethylene (HDPE) beads (diameter = 3 to 4 mm) using supercritical impregnation and catalyzed hydrogenation of numerous aromatic compounds in sc-CO_2_, including benzene and naphthalene [5]. The principles of synthesizing and using Pd/HDPE as a heterogeneous catalyst in sc-CO_2_ are based on the frequent swelling of polymers in sc-CO_2_[6-8]. CO_2_-soluble metal precursors can diffuse into the CO_2_-swollen polymers. Following hydrogen reduction, metal ions in the precursors are reduced to zero-valence metals and aggregate until becoming nanoparticles resulting from steric confinement by the polymer structure. The metal/polymer composites swell again in sc-CO_2_, allowing CO_2_-soluble reactants to diffuse into polymer structures that contact embedded metal nanoparticles for catalytic reactions. The products, catalysts, and sc-CO_2_ are separated by depressurization. The examples of using Pd/HDPE as a heterogeneous catalyst in sc-CO_2_ are catalytic hydrodechlorination and hydrogenation of polychlorinated biphenyl (PCB). Under mild conditions (P_CO2_ = 100 to 200 atm, P_H2_ = 10 atm, and temperature = 40 to 100°C), PCBs can be converted to bicyclohexyl efficiently using consecutive reactions in sc-CO_2_ with static and dynamic modes [9-12]. The catalytic system fulfills green chemistry principles and economic requirements for developing advanced industrial processes.

The dispersion of porous heterogeneous catalysts in the fluid phase, external mass transfer adjacent to the catalysts, and internal mass transfer within the catalysts are three key catalyst performance factors [13]. The uniform dispersion of catalysts and alleviation of external mass transfer resistance in a catalytic system are typically achieved by vigorous stirring during the fluid phase when the influence of internal mass transfer resistance is lowered by reducing catalyst support size to a specific value (e.g., diameter = approximately 100 μm) [14]; however, handling and recovering micro-powdered catalysts is not simple. Moreover, uniform dispersion of catalysts in a reactor is usually difficult to achieve by stirring, and the aggregation of numerous catalysts may affect their performance.

In this study, a solvent-free approach involving supercritical foaming and supercritical impregnation is used to prepare recyclable and geometrically variable microcellular high-density polyethylene-stabilized palladium nanoparticles (Pd/m-HDPE) with uniform particle dispersion and no significant mass transfer resistance in sc-CO_2_. Supercritical foaming is used to prepare microcellular polymer foams for numerous applications such as absorbents, biomedical materials, and lightweight structural devices [15-18]. The pore size and density can be altered by controlling pressure, temperature, depressurization rate, and other factors.

## Methods

### Materials and equipments

HDPE beads, palladium(II) hexafluoroacetylacetonate, hexane, and biphenyl were purchased from Aldrich (Milwaukee, WI, USA). All chemicals were used as received. Scanning electron microscopy (SEM) (Hitachi S-2400, Tokyo, Japan), transmission electron microscopy (TEM) (Hitachi H-7100), atomic absorption (AA) (Perkin Elmer AAnalyst 100, Waltham, MA, USA), ultraviolet-visible (UV/Vis) spectroscopy (GENESYS 10 S, ThermoSpectronic, Rochester, NY, USA) and gas chromatography flame ionization detection (GC/FID) (Varian CP-3800, Palo Alto, CA, USA) are used in this study.

### Preparation of recyclable and geometrically variable pd/m-HDPE catalysts

The synthesis procedure for preparing recyclable and geometrically variable Pd/m-HDPE catalysts involves supercritical foaming, supercritical impregnation, and hydrogen reduction. The experimental setups for catalyst synthesis and catalytic reactivity test are described in previous literature [9,10]. A beaker filled with 2.5 g of white HDPE beads (circular flat white beads with an average diameter, height, and weight of 3 mm, 1.5 mm, and 14 mg, respectively; T_m_ = 130°C) is placed in a 20-mL high-pressure reactor at 140°C and pressurized with 200 atm of CO_2_ for the supercritical foaming process. After 3 h, the reactor was depressurized completely in 10 s. A small section is removed from the top of the resulting m-HDPE to produce a level surface. Subsequently, 100 mg of yellow palladium (II) hexafluoroacetylacetonate, Pd(hfa)_2_, and a microcellular HDPE support were placed into the high-pressure reactor at 90°C and pressurized with 100 atm of CO_2_ for the supercritical impregnation process. After 3 h, the reactor was depressurized. The depressurization time was less than 1 min to avoid impregnated Pd(hfa)_2_ diffusing outside of the m-HDPE support. The Pd(hfa)_2_-impregnated HDPE support, the reactor, and the tubing were cleaned using hexane to recover Pd(hfa)_2_ outside the support for reuse. The cleaned Pd(hfa)_2_-impregnated HDPE support was then placed into the reactor again, and the Pd^2+^ in the precursors was reduced to a zero-valence state with 10 atm of hydrogen gas in 200 atm of sc-CO_2_ at 90°C. The resulting Pd/m-HDPE catalyst was cleaned for 1 h with sc-CO_2_ to remove impurities. The conversion variation test to compare agitation speed or catalyst volume was performed as follows: a small beaker containing 10 μL of biphenyl stock solution in hexane (concentration = 7.74 × 10^-2^ M) was placed on top of Pd/m-HDPE catalysts in a 20-mL high-pressure reactor. Following hexane evaporation and achieving the required temperature (50°C), the reactor was pressurized with CO_2_ at 100 atm to dissolve biphenyls in sc-CO_2_ in advance. In total, 200 atm of CO_2_ containing 10 atm of H_2_ was injected into the reactor from a 40-mL H_2_ storage cell. After 5 min, the system outlet valve was opened, and the effluent was collected in 10 mL of hexane for UV/Vis and GC/FID analysis. The collection process was continued for 30 min with a flow rate of 1 mL min^-1^ using identical experimental conditions (200 atm of CO_2_ at 50°C) to recover all reactants and products. The catalysts and high pressure system were cleaned for another 30 min following the collection process.

## Results and discussion

### Preparation of microcellular high-density polyethylene-stabilized pd nanoparticles (pd/m-HDPE)

**Figure 1:**
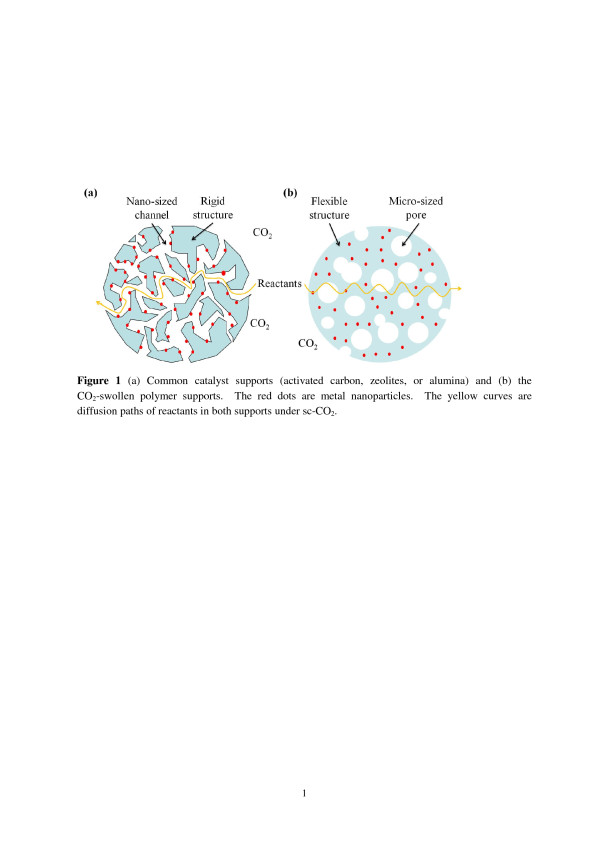
**The micrometer-sized catalyst supports and CO**_**2**_**-swollen centimeter-sized polymer supports.** (**a**) Common micrometer-sized catalyst supports (activated carbon, zeolites, or alumina) and (**b**) the CO_2_-swollen centimeter-sized polymer supports. The red dots are metal nanoparticles. The yellow curves are diffusion paths of reactants in both supports under sc-CO_2_.

The concept of creating Pd/m-HDPE for heterogeneous catalysis in sc-CO_2_ is described as follows. The diffusion of molecules in porous materials is highly dependent on the pore sizes [13]. Three diffusion types are in porous materials: molecular diffusion, Knudsen diffusion, and single-file diffusion. For gas-phase molecules, the approximate pore sizes for each regime are >100 nm, 10 to 100 nm, and <10 nm. Their respective diffusion coefficients are >10^-2^, 10^-2^ to 10^-4^, and <10^-4^ cm^2^s^-1^. The pore sizes of common high surface area catalyst supports (activated carbon, zeolites, and alumina) are usually nanosized and are thus unfavorable for molecular diffusion because their supports are too rigid for molecules to pass through their structures. Molecules must travel via nanosized pore channels to make contact with metal nanoparticles adsorbed on the surface of the pores for catalytic reactions (Figure 1a). Consequently, small-volume catalysts in the micrometer range are usually applied to reduce internal mass transfer resistance. One example is the hydrodechlorination of 2-chlorobiphenyl over sulfide Ni-Mo/γ-Al_2_O_3_ in hexadecane at 573 K and 2 MPa of H_2_[14]. Significant internal mass transfer resistance is observed in 3-mm alumina-supported catalysts. When the catalyst diameter is reduced from 3 mm to 100 μm, the hydrodechlorination rate is increased by 4.5 times because the mean reactant diffusion path from the catalyst surface to active sites within the catalyst is reduced. Nevertheless, uniform dispersion and recovery of these powdered catalysts are difficult. To use large-volume catalysts in the millimeter or centimeter range that can be recycled and handled easily, and is more favorable to industrial applications, expanding the pore size must be applied to promote molecular diffusion in the porous structure. However, this measure causes less metal nanoparticle surface area support in a fixed amount of catalysts. One potential method to solve this issue is to use microcellular polymers as metal nanoparticle supports for reactions in sc-CO_2_ (Figure 1b).

Metal nanoparticles are tangled by flexible polymer chains. Under sc-CO_2_, polymers are swelled, and the mobility of polymer chains is enhanced significantly, allowing molecules to pass through their structures. The creation of microcellular pores further facilitates the molecule diffusion in the polymer matrix. Consequently, molecules can theoretically approach each embedded metal nanoparticle from all directions through microcellular pores and flexible swollen polymer structures. Furthermore, nanoparticles may detach from the pore surface and block nanosized pore channels during reaction, recovery, and cleaning processes, causing catalyst deactivation in the case of common supports (Figure 1a). Such polymer support issues can be prevented because nanoparticles are surrounded and sterically confined by numerous polymers. The possibility of metal nanoparticle leaching should be minimized.

The supercritical foaming process, temperature, CO_2_ pressure, and depressurization rate are three parameters that can tune the pore size and density of HDPE easily. Previous research on foaming of polystyrene and poly (D,L-lactic acid) using sc-CO_2_ (Tsivintzelis et al.) shows that pore density increases and pore size decreases with decreasing temperature, thereby increasing CO_2_ pressure and the depressurization rate [18].

**Figure 2:**
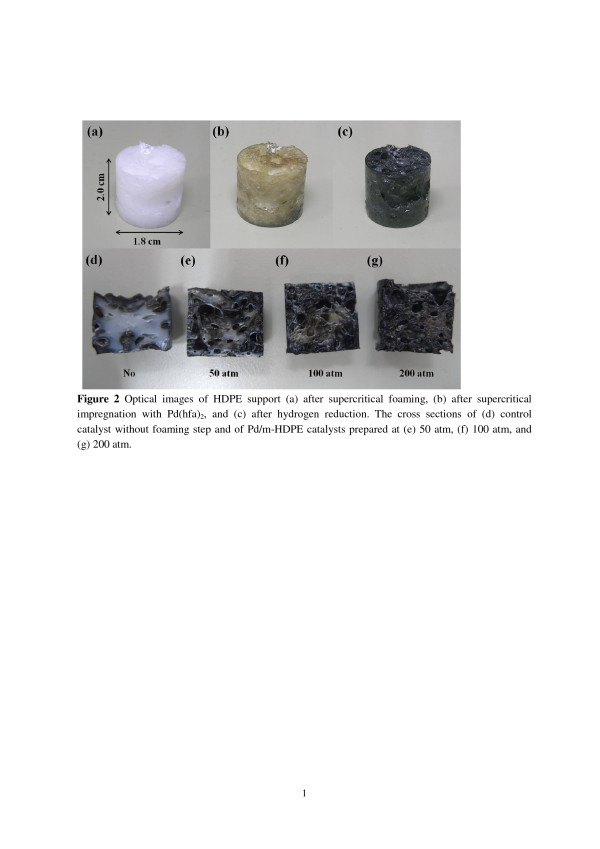
**The optical images of HDPE support.** Optical images of HDPE support (**a**) after supercritical foaming, (**b**) after supercritical impregnation with Pd(hfa)_2_, and (**c**) after hydrogen reduction. The cross sections of (**d**) control catalyst without foaming step and of Pd/m-HDPE catalysts are prepared at (**e**) 50, (**f**) 100, and (**g**) 200 atm.

In this study, the foaming temperature is set at 140°C, which is slightly higher than the melting point (approximately 130°C) of HDPE beads. No HDPE foaming is observed if the temperature is below 130°C, whereas higher temperatures (>140°C) result in a decrease in CO_2_ density, thereby causing low pore density and large pore size. The depressurization process is controlled as short as possible, in which 10 s was used to reach atmosphere pressure from supercritical fluid conditions. Various CO_2_ pressures (50, 100, and 200 atm) were tested to determine the influence of resulting pores on molecule diffusion in the microcellular HDPE structure. The experimental conditions for supercritical impregnation of Pd(hfa)_2_ into HDPE and hydrogen reduction are similar to those reported in previous literature [9]. Supercritical foaming and impregnation are not combined in one process because Pd^2+^ of Pd(hfa)_2_ undergoes gradual thermal reduction if temperatures exceed 90°C. Additionally, larger Pd nanoparticles with lower surface-to-volume ratios could be generated by HDPE structures with more flexibility when the temperature exceeds T_m_ of HDPE. Figure 2 shows optical images of HDPE supports following each process. Over 100 pieces of the original HDPE beads formed a large cylinder (height = 2.0 cm; diameter = 1.8 cm, 2a) with small pores following supercritical foaming. The cylinder became yellowish when the supercritical impregnation of Pd(hfa)_2_ into the cylinder was completed, indicating the existence of Pd(hfa)_2_ (Figure 2b). The yellow cylinder became black following hydrogen reduction, indicating the formation of Pd nanoparticles inside the cylinder (Figure 2c). The characterization of the pore sizes and metal nanoparticles by SEM and TEM are discussed in the following paragraph.

For comparison, a control catalyst resembling the original Pd/HDPE catalysts [5] was prepared following the same mentioned procedure, except for the supercritical foaming process. The control catalyst and Pd/m-HDPE catalysts synthesized under different CO_2_ pressures were then halved to observe their interior structure. Figure 2d shows the control catalyst cross section. Only the surface (approximately 1 mm) turned black and the rest remained white, indicating that the sc-CO_2_ swelling phenomenon cannot impregnate Pd(hfa)_2_ deep into the HDPE structure when the HDPE support is increased from millimeter [5] to centimeter range. The cross section of three Pd/m-HDPE catalysts prepared at 50, 100, and 200 atm (Figures 2e-g) were almost completely black. Increasing CO_2_ pressure caused a decrease in pore sizes, an increase in pore density, and an increase in Pd/m-HDPE volume at a fixed temperature because of the increase in pore density. The remaining white area following the hydrogen reduction process is described by the following: no foaming > 50, >100, and > 200 atm. The visual observation indicates that the microcellular structure created by supercritical foaming enhances the internal mass transfer of molecules in HDPE support significantly and Pd/m-HDPE catalysts prepared at the CO_2_ pressure of 200 atm appear to possess the most efficient molecule diffusion within its structure. Higher CO_2_ pressure was not tested because of safety reasons, although it may achieve a more significant result with respect to internal mass transfer.

### Characterization of microcellular pd/HDPE

**Figure 3:**
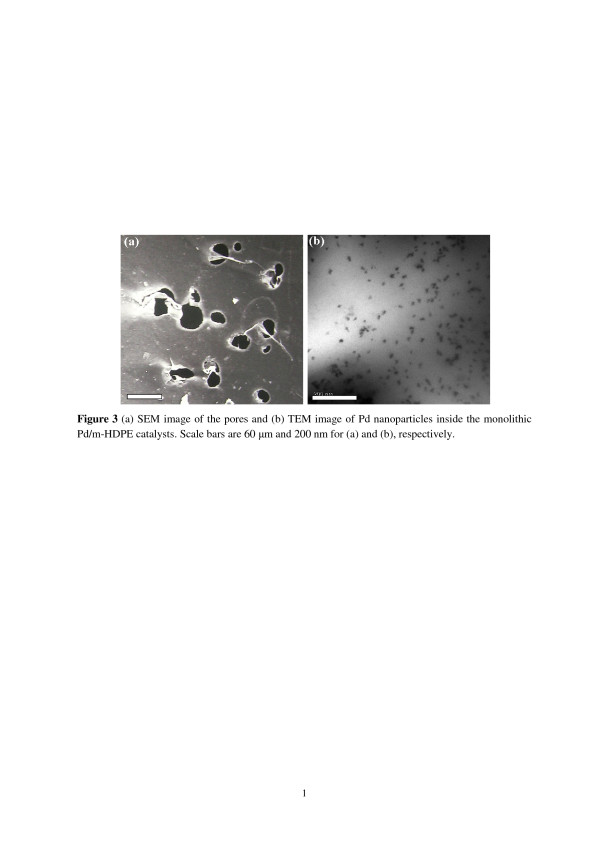
**The SEM image of the pores and TEM image of the Pd nanoparticles.** (**a**) SEM image of the pores and (**b**) TEM image of Pd nanoparticles inside the monolithic Pd/m-HDPE catalysts. Scale bars are 60 μm and 200 nm for (**a**) and (**b**), respectively.

Figure 3a shows the SEM image of pores within the Pd/m-HDPE catalysts. Micropores (approximately 20 to 60 μm) created by supercritical foaming are on/within the HDPE support. These pores are not blocked by Pd particles. Miller and Kumar. demonstrated the preparation of microcellular HDPE foams using a subcritical CO_2_ process [19]. The resulting HDPE structure has micro-sized cells with interiors fabricated by interconnected open nanosized pores. The average cell size is 67 μm at a CO_2_ saturation pressure of 300 atm and foaming temperature of 150°C. Figure 3b shows a TEM image of Pd particles confined within an HDPE structure. Based on a 50 particle count, the Pd particles with irregular shapes and an average diameter of 10.5 ± 3.5 nm are randomly dispersed inside the HDPE support. The nanoparticles are slightly larger than those reported by Ohde et al. (between 2 and 10 nm) in which Pd/HDPE catalysts were prepared only with supercritical impregnation and hydrogen reduction at 200 atm of CO_2_ at 50°C [5]. Yuan and Marshall have shown that low Pd(hfa)_2_ concentrations result in smaller and more spherical Pd nanoparticles, whereas high Pd(hfa)_2_ concentrations generate larger Pd nanoparticles with irregular shapes [20]. Supercritical foaming processes that change the HDPE structure may also contribute to variations in the size and shape of Pd nanoparticles in m-HDPE. These Pd nanoparticles are in their zero-valence state according to XPS data [9]. AA analysis (Perkin Elmer AAnalyst 100) shows that Pd concentration in the HDPE support is approximately 426 ppm after dry ashing and acid digestion (HCl) of Pd/m-HDPE [21,22]. The concentration is similar to that in a previous study (average concentration = 430 ppm) that analyzed Pd/HDPE using neutron activation analysis [9].

**Figure 4:**
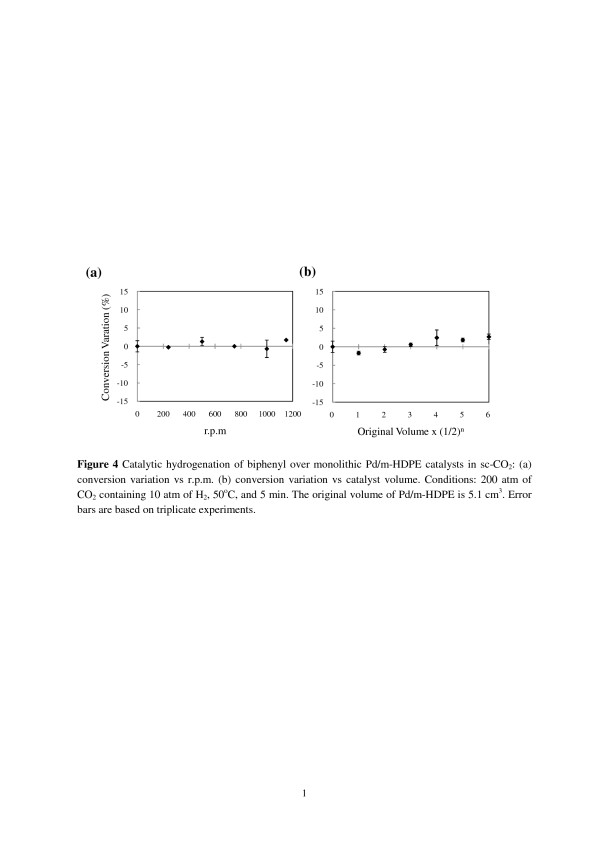
**Catalytic hydrogenation of biphenyl over monolithic Pd/m-HDPE catalysts in sc-CO**_**2**_**.** (**a**) Conversion variation vs rpm and (**b**) conversion variation vs catalyst volume. Conditions, 200 atm of CO_2_ containing 10 atm of H_2_, 50°C, and 5 min. The original volume of Pd/m-HDPE is 5.1 cm^3^. Error bars are based on triplicate experiments.

In the absence of external and internal mass transfer, resistances in a catalytic system can be evaluated approximately by the influence of the stirring speed and the catalyst size on the conversion of catalytic reactions if a constant conversion is observed [13]. Hydrogenation of biphenyls over Pd/m-HDPE catalysts in sc-CO_2_ was tested for this purpose because biphenyl is related to our PCB remediation method development [9-12]. The products were collected in hexane trap solutions and analyzed by UV/Vis spectroscopy and GC/FID to determine the conversion (%) of biphenyls. Figure 4a shows the conversion variation (%) versus rpm plot for 200 atm of CO_2_ containing 10 atm of H_2_ obtained at 50°C and 5 min over 5.1 cm^3^ of a Pd/m-HDPE catalyst, as shown in Figure 2c. Two products, cyclohexylbenzene and bicyclohexyl, are observed. The conversion variation under this condition without stirring is 37.8% (selectivity, 92.4% to cyclohexylbenzene, 7.6% to bicyclohexyl). Increasing rpm does not increase biphenyl conversion, showing the absence of external mass transfer resistance in the catalytic system resulting from high diffusivity, low viscosity, and low sc-CO_2_ surface tension, as well as the uniform dispersion of Pd nanoparticles embedded in HDPE support resembling a homogenization of heterogeneous catalysis. Figure 4b shows the conversion variation (%) versus catalyst volume plot obtained under the same conditions as the stirring speed test. The conversion variation is almost constant with decreasing catalyst volume, implying a nonsignificant internal mass transfer resistance within Pd/m-HDPE catalysts because of the CO_2_-swollen HDPE support and the microcellular structure contributing to the facile inner mass transfer.

### Reusability of pd/m-HDPE catalysts

No reduction in conversion was observed following more than 50 trials of reusability of Pd/m-HDPE catalysts. Following each experiment, the catalyst was cleaned with sc-CO_2_ for 30 min with a flow rate of 1 mL/min under the same experimental conditions, which is relatively simple compared with other catalyst cleaning procedures. Unlike powdered catalysts that lose or block and contaminate the experimental equipment during reaction and recovery processes, the monolithic Pd/m-HDPE catalyst is free of these problems. Furthermore, the HDPE supports with simple molecular structures that contain only C and H are non-toxic, economical, do not absorb molecules easily, and may cause difficulty in product recovery and catalyst cleaning.

### Other advantages of pd/m-HDPE heterogeneous catalyst

The preliminary experimental data demonstrate that supercritical foaming and impregnation can be used as a solvent-free technique to prepare Pd/m-HDPE as a heterogeneous catalyst for catalytic hydrogenation in sc-CO_2_ without significant mass transfer resistance. Compared with other sc-CO_2_ catalysts, the advantages of these Pd/m-HDPE catalysts include a) the pre-dispersion of metal nanoparticles in the polymer matrix that resembles a homogeneous system and, consequently, does not require vigorous stirring to achieve uniform dispersion; b) the microcellular HDPE support structure coupled with the sc-CO_2_ polymer swelling property allows simple reactant diffusion inside the polymers, facilitating internal mass transfer; c) large sizes and various shapes of catalysts can be prepared, simplifying experimental or industrial design and operation; d) the catalysts are recyclable and reusable without complex recovery and cleaning procedures; and e) the fabrication of Pd/m-HDPE catalysts using this method is in a closed system; thus, the potential hazards of nanoparticles to the environment resulting from system leakage is avoidable.

## Conclusions

By taking advantage of sc-CO_2_ characteristics, a green solvent and microcellular high-density polyethylene-stabilized palladium nanoparticles are successfully developed as heterogeneous catalysts in sc-CO_2_. In a large cylinder (2.0 cm height × 1.8 cm diameter) with pore sizes between 20 and 60 μm, Pd nanoparticles with an average diameter of 10.5 ± 3.5 nm are randomly dispersed. The conversion variations are almost constant with increasing agitation speed or decreasing catalyst volume, implying the presence of nonsignificant external and internal mass transfer resistance on the catalyst surface and within the catalyst structure. The CO_2_-swollen microcellular HDPE support enhances molecule diffusion inside its structure significantly, facilitating heterogeneous catalytic reactions. The findings in this study show novel advances in the field of heterogeneous catalysis, as well as potential application in environmental remediation. The presented supercritical techniques may serve as a simple and environmentally friendly approach for the preparation of various microcellular-polymer-stabilized metal nanoparticles for heterogeneous catalytic reactions in supercritical fluids. Additionally, as the field of nanotechnology expands into consumer markets, manufacturing nanoparticles by using efficient, low-cost approaches is essential, one which must be socially responsible to minimize their environmental impact on the environment.

## Competing interests

The authors declare that they have no competing interests.

## Authors’ contributions

WS carried out the nanocomposite preparation and characterization, designed the concept and experiment, analyzed the results and drafted, revised and finalized the manuscript. BZ participated in the reusability of Pd/m-HDPE catalysts and in the experiment data analysis. HY and JJ contributed to the preparation and discussion of the experimental data analysis and preparation of the figures. KH integrated the research, revised, and finalized the manuscript. All authors read and approved the final manuscript.
